# Prevalence of hepatitis A across various countries in the Middle East, African and Eastern European countries

**Published:** 2016

**Authors:** Abbolmajid Ghasemian

**Affiliations:** 1 Department of Bacteriology, Faculty of Medical Sciences, Tarbiat Modares University, Tehran, Iran.; 2 Department of Epidemiology, Pasteur Institute of Iran, Tehran, Iran

**Keywords:** Hepatitis A, Middle East Region, North Africa, Eastern Europe

**Dear Editor,**

Hepatitis A virus (HAV) is highly contagious and causes liver disease that is symptomatic in more than 80% of adults besides children. The virus is widespread in all parts of the world and transmitted by the fecal-oral route (1). The infection is endemic in Africa except in South Africa. Moreover, Eastern Europe, Asia and Africa are at high risk of infection. Among the North African countries, Egypt has the lowest rate of antibody against virus, and likewise among the Middle East countries, Saudi Arabia, Syria and Turkey have lower prevalence of specific antibody. In a review, hepatitis A seroprevalence in Tehran, Golestan and Hormozgan cities of Iran was 85%, 99%, and 96%, respectively. Moreover, the overall seroprevalence of virus in the general population of these provinces was 86% and did not differ between the males and females. On the other hand, the prevalence in younger individuals and in urban populations was lower 70% (2). Among Kurdish refugees from Turkey and Iraq, the prevalence of anti- hepatitis A was 94.4%, showing a high rate of viral hepatitis enterically transmitted in Kurds (3). In Pakistan, 2 of 109 patients had anti-HAV IgG (4). In another survey in Saudi Arabia from 1214 patients, 10% were positive for HAV (5). A high prevalence of HAV antibody was detected among participants in Aden province, Yemen with 86.6%, and it has been uncovered that viral hepatitis is a major problem in this area as an endemic disease (6). In a study in Iraq, the anti-HAV IgM antibody was 41.0% among rural and urban blood donors and it has been estimated that the hepatitis A-IgG antibodies in Iraqi population is about 96.4% in 2011 (7). 

Rural areas of Egypt have a high prevalence of HAV, reaching to 100% (8). Consumption of village water, contamination of drinking water sources by sewage and the use of indoor dry pits are the major risk factors for HAV transmission in rural Egypt. In Libya, similar to Egypt, most HAV infections are acquired since childhood. The HAV antibodies have been detected among 60–70% of three years old children, and reaches to 100% in 7 years old children (9). Several studies have shown high HAV prevalence in Tunisia at 84.0-92% and there is no national program for viral hepatitis in this country (10-12). Several outbreaks of HAV infection were erupted among children tourists from European countries, and thus vaccination in this situation seems essential among travelers (13-15). In Algeria, there is a high prevalence of the disease, where 96% of individuals are anti-HAV antibodies positive with more symptoms among young children (16). Morocco is an intermediate endemic area for the infection and anti-HAV antibodies vary between 45% and 70% in children less than 6 years old and those between 7–14 years old, respectively (17).

## References

[1] Mayer CA, Nelson AA (2010). Hepatitis A: prevention in travellers. Aust Fam Phys.

[2] Merat S, Rezvan H, Nouraie M (2010). Seroprevalence and risk factors of hepatitis A virus infection in Iran: a population based study. Arch Iran Med.

[3] Chironna M, Germinario C, Lopalco P (2003). Prevalence rates of viral hepatitis infections in refugee Kurds from Iraq and Turkey. Infection.

[4] Bryan JP, Iqbal M, Tsarev S (2002). Epidemic of hepatitis E in a military unit in Abbotrabad, Pakistan. Am J Trop Med Hyg.

[5] Al-Tawfiq JA, Anani A (2008). Profile of viral hepatitis A, B, and C in a Saudi Arabian hospital. Med Sci Monit.

[6] Bawazir AA, Hart CA, Sallam TA, Parry CM, Beeching NJ, Cuevas LE (2010). Seroepidemiology of hepatitis A and hepatitis E viruses in Aden, Yemen. Trans R Soc Trop Med Hyg.

[7] Turky AM, Akram W, Al-Naaimi AS, Omer AR, Al-Rawi JR (2011). Analysis of acute viral hepatitis (A and E) in Iraq. Global J Health Sci.

[8] Meky FA, Stoszek SK, Hamid MA (2006). Active surveillance for acute viral hepatitis in rural villages in the Nile Delta. Clin Infect Dis.

[9] Kamal SM, Mahmoud S, Hafez T, EL-Fouly R (2010). Viral hepatitis A to E in South mediterranean countries. Mediterr J Hematol Infect Dis.

[10] Rezig D, Ouneissa R, Mhiri L (2008). Seroprevalences of hepatitis A and E infections in Tunisia. Pathol Biol.

[11] Gharbi‐Khelifi H, Sdiri K, Ferre V (2007). A 1‐year study of the epidemiology of hepatitis A virus in Tunisia. Clin Microbiol Infect.

[12] Letaief A, Kaabia N, Gaha R (2005). Age-specific seroprevalence of hepatitis a among school children in central Tunisia. Am J Trop Med Hyg.

[13] Pröll S, Nothdurft H (2004). The risk of contracting hepatitis A or hepatitis B run by visitors to the Mediterranean and Eastern Europe. MMW Fortschritte der Medizin.

[14] FitzSimons D, Hendrickx G, Vorsters A, Van Damme P (2010). Hepatitis A and E: update on prevention and epidemiology. Vaccine.

[15] Arya SC, Agarwal N (2010). Re: Hepatitis A and E: Update on prevention and epidemiology. Vaccine.

[16] Smahi M, Rahmoun L, Ghomari S (2009). Seroprevalence and risk factors of hepatitis A among children in Tlemcen (north-west Algeria). Arch Pediatr.

[17] Bouskraoui M, Bourrous M, Amine M (2009). Prevalence of anti-hepatitis A virus antibodies in children in Marrakech. Arch Pediatr.

